# Traditional Uses, Bioactive Chemical Constituents, and Pharmacological and Toxicological Activities of *Glycyrrhiza glabra* L. (Fabaceae)

**DOI:** 10.3390/biom10030352

**Published:** 2020-02-25

**Authors:** Gaber El-Saber Batiha, Amany Magdy Beshbishy, Amany El-Mleeh, Mohamed M. Abdel-Daim, Hari Prasad Devkota

**Affiliations:** 1 National Research Center for Protozoan Diseases, Obihiro University of Agriculture and Veterinary Medicine, Nishi 2-13, Inada-cho, Obihiro 080-8555, Hokkaido, Japan; amanimagdi2008@gmail.com; 2 Department of Pharmacology and Therapeutics, Faculty of Veterinary Medicine, Damanhour University, Damanhour 22511, AlBeheira, Egypt; 3 Department of Pharmacology, Faculty of Veterinary Medicine, Menoufia University, Menofia Governorate 32511, Egypt; Amany.ahmed1074@gmail.com; 4 Department of Zoology, College of Science, King Saud University, P.O. Box 2455, Riyadh 11451, Saudi Arabia; abdeldaim.m@vet.suez.edu.eg; 5 Pharmacology Department, Faculty of Veterinary Medicine, Suez Canal University, Ismailia 41522, Egypt; 6 Graduate School of Pharmaceutical Sciences, Kumamoto University, 5-1 Oe-Honmachi, Chuo-ku, Kumamoto City 862-0973, Kumamoto, Japan; devkotah@kumamoto-u.ac.jp

**Keywords:** *Glycyrrhiza glabra*, herbal remedies, pharmacological activities, phytoconstituents, pharmacokinetics

## Abstract

Traditional herbal remedies have been attracting attention as prospective alternative resources of therapy for diverse diseases across many nations. In recent decades, medicinal plants have been gaining wider acceptance due to the perception that these plants, as natural products, have fewer side effects and improved efficacy compared to their synthetic counterparts. *Glycyrrhiza glabra* L. (Licorice) is a small perennial herb that has been traditionally used to treat many diseases, such as respiratory disorders, hyperdipsia, epilepsy, fever, sexual debility, paralysis, stomach ulcers, rheumatism, skin diseases, hemorrhagic diseases, and jaundice. Moreover, chemical analysis of the *G. glabra* extracts revealed the presence of several organic acids, liquirtin, rhamnoliquirilin, liquiritigenin, prenyllicoflavone A, glucoliquiritin apioside, 1-metho-xyphaseolin, shinpterocarpin, shinflavanone, licopyranocoumarin, glisoflavone, licoarylcoumarin, glycyrrhizin, isoangustone A, semilicoisoflavone B, licoriphenone, and 1-methoxyficifolinol, kanzonol R and several volatile components. Pharmacological activities of *G. glabra* have been evaluated against various microorganisms and parasites, including pathogenic bacteria, viruses, and *Plasmodium falciparum*, and completely eradicated *P. yoelii* parasites. Additionally, it shows antioxidant, antifungal, anticarcinogenic, anti-inflammatory, and cytotoxic activities. The current review examined the phytochemical composition, pharmacological activities, pharmacokinetics, and toxic activities of *G. glabra* extracts as well as its phytoconstituents.

## 1. Introduction

Recently, antibiotics and most drugs on the market have shown unwanted symptoms and the emergence of resistant pathogenic microorganisms, toxic effects related to these drugs, and withdrawal issues restricting their use in many countries [1,2]. Therefore, research on herbal plants has provided modern medicine with several useful chemical ingredients that have been used to manage various ailments. However, many people in developing countries, especially in Africa and Asia, still rely on crude herbal extracts to treat several human and animal ailments [3,4]. This is partly because these extracts are inexpensive and easily accessible. Many plant species have been reported to have pharmaceutical activities due the presence of several bioactive components like glycosides, saponins, flavonoids, steroids, tannins, alkaloids, and terpenes [5,6,7]. To date, medicinal plants have been documented as an important source for discovering new pharmaceutical molecules that can been used to treat serious diseases [5,8]. For instance, Batiha et al. [9] as well as Beshbishy et al. [10] reported the antiprotozoal activity of chalcones and ellagic acid, the naturally-derived phytoconstituents isolated from herbal extracts against *Plasmodium*, *Leishmania*, *Trypanosoma*, *Babesia*, and *Theileria* parasites. Moreover, phenolic and flavonoid compounds exhibited antioxidant, anticancer, anti-inflammatory, and antidiabetic activities [11].

*Glycyrrhiza glabra* L. (Family: Fabaceae) (Table 1) is a small perennial herb, commonly known as licorice, sweet wood, or mulaithi, that is indigenous to Eurasia, northern Africa, and western Asia [12]. The *Glycyrrhiza* genus is widely distributed worldwide and it consists of more than 30 species. Its name was obtained from the Grecian words glykys, which means sweet, and rhiza, which means root, while the glabra species name refers to the smooth husks and is acquired from the Latino word glaber that implies bare or slick [13]. *G. glabra* is a 1 m tall herbaceous plant that consists of 9–17 leaflets and 7–15 cm long pinnate leaves, with pale whitish blue to purple flowers with a length ranging from 0.8 to 1.2 cm. The fruits are 2–3 cm long oblong pods, containing several seeds with stoloniferous roots [14]. Licorice grows near a river or stream in fertile, clay, or sandy soil, where there is water available for the plant to flourish [15]. Rhizomes and roots are the most important medicinal parts of licorice that have been reported to be used alone or with other herbs for the treatment of many digestive system disorders (e.g., stomach ulcers, hyperdipsia, flatulence, and colic), respiratory tract disorders, such as coughs, asthma, tonsillitis, and sore throat, epilepsy, fever, sexual debility, paralysis, rheumatism, leucorrhoea, psoriasis, prostate cancer, malaria, hemorrhagic diseases, and jaundice. Moreover, it can be used as a food and beverage flavoring agent and added to flavor tobacco products [15].

## 2. Physicochemical Features

Physicochemical examination of *G. glabra* roots documented that chloroform, petroleum ether, n-butanol, and methanol extract yields were 4.67 ± 0.23%, 10.56 ± 1.53%, 6.54 ± 0.84%, and 13.89 ± 2.42%, respectively, while acid insoluble ash, total, and water-soluble ash values were 0.56 ± 0.34%, 4.67 ± 0.35%, and 6.54 ± 0.22%, respectively [16].

### 2.1. Chemical Constituents

*Glycyrrhiza glabra* L. roots contain several active compounds (Table 2), including flavonoids, such as liquirtin, rhamnoliquirilin, liquiritigenin, prenyllicoflavone A, glucoliquiritin apioside, 1-metho-xyphaseolin, shinpterocarpin, shinflavanone, licopyranocoumarin, glisoflavone, licoarylcoumarin, and coumarin-GU-12, and saponins, namely, glycyrrhizin (60-times more sugary than sugarcane). In addition, four isoprenoid-substituted phenolic constituents (isoangustone A, semilicoisoflavone B, licoriphenone, and 1-methoxyficifolinol), kanzonol R (prenylated isoflavan derivative) and several volatile components (pentanol, tetramethyl pyrazine, hexanol, terpinen-4-ol, linalool oxide A and B, geraniol, and α-terpineol) have also been reported. Whereas propionic acid, 1-methyl-2-formylpyrrole, 2,3-butanediol, benzoic acid, ethyl linoleate, furfuryl formate, trimethylpyrazie, furfuraldehyde, methyl ethyl ketone, and maltol were isolated from the essential oil. Glycyrrhizin, a saponin compound, as well as its aglycone glycyrrhetinic acid, are the potent components in *G. glabra*. Glycyrrhizin consists of glycyrrhetic acid and triterpenoid aglycone, associated with glucuronic acid disaccharide, and it can be found naturally as calcium and potassium salts in licorice root [17,18,19]. In humans, glycyrrhizin can be metabolized and converted to glycyrrhetinic acid and, thus, the pharmacological activities of glycyrrhizin are similar to those of glycyrrhetinic acid [12].

Raw and tea licorice infusions contains protein, fat, moisture, raw ash, fiber, silica, carbohydrates, minerals (calcium, phosphorus, sodium, potassium, zinc, and copper), and amino acids, including serine, aspartic, glycine, glutamic, threonine, valine, prolinealanine, isoleucine, tyrosine, leucine, lysine, phenylalanine, tyrosine, and histidine. Interestingly, HPLC analysis of the methanolic extract of licorice detected the presence of several organic acids, such as acetic, propanoic, fumaric, citric, butyric, malic, and tartaric acids [20].

### 2.2. Mechanisms of Action

The diverse pharmacological activities of licorice and its related compounds are due to its various mechanisms of action. For instance, the *Glycyrrhiza* genus is well known as an 11 beta-hydroxysteroid dehydrogenase (11β-HSD2) inhibitor that subsequently inhibits cortisol inactivation, leading to an increase in the mineralocorticoid efficacy or pseudohyperaldosteronism. Pseudohyperaldosteronism of licorice is mainly due to the presence of glycyrrhetinic acid that acts by two different mechanism of actions: either by inhibiting 11β-HSD2, which binds directly to the mineralocorticoid receptor as an agonist, or it can be reversed by coincubation with the mineralocorticoid receptor blocker and spironolactone derivative, canrenone, which was determined by radioreceptor test in human mononuclear leukocytes (MNL) [21]. The inhibitory effect of glycyrrhetinic acid on 11HSD2 occurs even at low serum concentrations, while its binding to mineralocorticoid receptor appears later, after it has been accumulated in the blood. Interestingly, Calò et al. [22] investigated the inflammatory effect of glycyrrhetinic acid and aldosterone using MNL. They revealed that mononuclear cells incubation with glycyrrhetinic acid and/or aldosterone improved the protein expression of the two inflammation markers, PAI-1 and p22phox, and this effect was reversed by coincubation with canrenone. The mineralocorticoid activity enhancement leads to high water and sodium reabsorption over potassium excretion, resulting in high blood pressure and the development of edema [23]. Notably, glycyrrhetinic acid and glycyrrhizin have been reported to restrict various RNA and DNA viruses’ growth, such as herpes simplex, herpes zoster, human immunodeficiency virus (HIV), and hepatitis B and C [24,25]. Moreover, they inhibited aldosterone hepatic metabolism and prevented the 5-β reductase activities in charge of the symptoms of well-known pseudoaldosterone [23]. *G. glabra* has been reported to display an anti-inflammatory activity similar to a steroid hormone (hydrocortisone) by inhibiting phospholipase A2 enzyme activity, which is crucial for various inflammatory processes. Moreover, an in vitro study demonstrated that glycyrrhizic acid suppresses the activity of cyclooxygenase and the formation of prostaglandin E2, preventing platelet aggregation indirectly [26]. The hepatoprotective and antioxidant activities of *G. glabra* and its phytoconstituents have been attributed to its efficacy in preventing reactive oxygen species (ROS) by neutrophils at the site of inflammation. Hispaglabridin A and B and isoflavones isolated from *G. glabra* extracts have been reported to prevent mitochondrial lipid peroxidation in rat liver cells caused by Fe sup 3+ in vitro. Phytochemicals isolated from *G. glabra* also exert their hepatoprotective efficacy via decreasing the serum liver enzyme levels and enhancing the tissue pathology in hepatitis patients [27].

## 3. Pharmacological Actions

### 3.1. Traditional Uses of G. glabra

Traditionally, licorice has been reported to treat many diseases, such as asthma, tonsillitis, sore throat, hyperdipsia, flatulence, epilepsy, fever, sexual debility, paralysis, coughs, stomach ulcers, heartburn, colic, swellings, rheumatism, skin diseases, acidity, leucorrhoea, bleeding, hemorrhagic diseases, and jaundice [28,29,30,31,32]. Moreover, it was traditionally used as an insecticide, laxative, anti-inflammatory, anti-ulcer, antibiotic, anti-arthritic, antiviral, memory stimulant due to its action as a monoamine oxidase (MAO) inhibitor, anti-cholinergic, antitussive, anti-caries, hypolipidemic, anti-mycotic, estrogenic, antioxidant, anticancer, and anti-diuretic agent [33]. It is used in the confection industry, such as in soft drinks, sweets, and alcohol as well as in the tobacco industry.

### 3.2. In Vitro Pharmacological/Biological Properties of G. glabra Extract and Its Metabolites

Previous reports documented the in vitro antitussive, expectorant, and demulcent activity of licorice powder and its extract. Pharmacologically, it was reported to treat bronchial cough, catarrh, and sore throat and these activities may be attributed to the existence of glycyrrhizin, which helps relieve congestion in the upper respiratory tract by accelerating the secretion of the bronchial mucosa [34,35]. Interestingly, *G. glabra* methanolic and flavonoid extracts have shown potent antibacterial effects toward *Bacillus subtilis, B. cereus, B. megaterium, Escherichia coli, Staphylococcus aureus, Enterococcus faecalis, Pseudomonas fluorescens, P. aeruginosa, Sarcina lutea, Salmonella paratyphi, S. typhi, Shigella boydii, S. dysenteriae, Vibrio parahaemolyticus*, and *V. mimicus* in vitro using the disc diffusion method [36,37]. Another in vitro study showed that methanolic *G. glabra* extract exhibited strong antibacterial efficacy toward all tested microorganisms except *P. aeruginosa*. However, flavonoids showed inhibitory activity against *S. aureus* and *E. faecalis*, but exhibited lower inhibitory activity against *P. aeruginosa* and *E. coli* [38]. In addition to that, the Kirby–Bauer test was employed to assess the antibacterial activities of chloroform, acetonic, ethyl acetate, and methanolic extracts of *G. glabra* against *S. typhimurium*, *B. coagulans*, *P. aeruginosa*, *S. aureus*, *E. faecalis*, and *E. coli* in vitro. The ethyl acetate, methanolic, chloroform, and acetonic extracts inhibited the growth of *S. typhimurium*, *E. coli*, and *B. coagulans* without affecting *S. aureus*, *P. aeruginosa*, and *E. faecalis* [39]. All *G. glabra* extracts inhibited the multiplication of the tested oral bacteria in vitro, while no strain revealed resistance to these extracts [40]. Moreover, the paper disc agar diffusion method was used to examine the in vitro antibacterial activity of aqueous and ethanolic *G. glabra* leave extracts in comparison with the activities of root extracts against *K. pneumoniae*, *E. coli*, *S. aureus, E. faecalis*, *B. subtilis*, *C. albicans*, and *P. aeruginosa*. The root and leaves extracts exhibited effectiveness against *C. albicans* and all examined Gram-positive bacteria in a dose-related pattern; however, the ethanolic leaves extract showed the highest effectiveness toward Gram-positive bacteria [41]. The antibacterial efficacy of glabridin towards Gram-negative and Gram-positive bacteria was registered and the highest efficacy was shown towards Gram-positive bacteria as well as H37Ra and H37Rv mycobacterial strains [42]. Additionally, Krausse et al. [43] reported the efficacy of glycyrrhetinic acid monoglucuronide acetylated (GAMG), glycyrrhetinic acid, and glycyrrhizic acid in vitro towards 29 strains of *Helicobacter pylori* and they revealed that glycyrrhetinic acid was the most effective compound by inhibiting 79.3% of the strains.

In addition to the above, the antiviral efficacy of *G. glabra* extracts and glycyrrhizic acid have been investigated against the multiplication of various viruses, including herpes simplex, Epstein–Barr, Human cytomegalovirus, hepatitis A, B, and C, Influenza, HIV, Varicella zoster, and severe acute respiratory syndrome (SARS) coronavirus [44]. Glycocoumarin, licopyranocoumarin, and licochalcone A exhibited growth inhibition of the giant cell structure in cell cultures infected with HIV without any cytotoxic activity [45]. Methanolic licorice extract exhibits potent anti-fungal effectiveness towards *Chaetomium funicola* M002 and *Arthrinium sacchari* M001 and this activity is due to the glabridin active compound [46]. Licochalcone A (a chalcone) has been documented to have potent antiplasmodial efficacy against chloroquine-susceptible (3D7) and chloroquine-resistant (Ddz) strains of *Plasmodium falciparum* in vitro [2,47]. Moreover, Christensen et al. [48] reported the in vitro antileishmanial efficacy of chalcones isolated from Chinese licorice roots, while Batiha et al. [49] exhibited the in vitro antipiroplasmic effect of chalcones against *Babesia* and *Theileria* parasites. 

Glycyrrhizin, deglycyrrhizinated licorice (DGL), as well as carbenoxolone isolated from licorice have shown antiulcer activity by suppressing gastrin secretion [50]. DGL is the processed form of licorice, after removal of the active compound glycyrrhizin, and was synthesized to avoid the side effects of licorice and complications caused by glycyrrhizin. It is available in wafers, capsules, liquid, and lozenges and its use has been documented in combination with antacids for the treatment of peptic ulcers [23]. Glycyrrhizin inhibits free radical reactions mediated by iron, free iron in hemoglobin, and carbonyl formation in hemoglobin that are manifested in diabetes. Hydromethanolic *G. glabra* root extract has been reported to have numerous polyphenolic compounds that revealed marked antioxidant efficacy in vitro and in vivo [51,52]. For instance, licochalcones B and D demonstrated their potential antioxidant efficacy by preventing microsomal lipid peroxidation and, thus, inhibiting red blood cells from oxidative hemolytic effects. Isoflavones (glabridin, 3′-hydroxy-4-O-methylglabridin, and hispaglabridin A) were also documented to possess potent antioxidant activity. Recently, isolated compounds, such as dehydro-stilbene derivatives have been documented as free radical scavengers [4,53]. Previous reports revealed that glycyrrhizin is broken down in the intestine and exhibits an anti-inflammation effect comparable with that of corticosteroid hormones, including hydrocortisone [54]. 

Glycyrrhizin is a famous anti-inflammatory component that has been documented to prolong thrombin and fibrinogen coagulation time and increase the duration of plasma recalcification in vitro and, accordingly, it is considered to be the first plant-based thrombin inhibitor. Glycyrrhizin was found to inhibit platelet aggregation caused by thrombin, while it did not affect the agglutination caused by collagen or platelet aggregating factor (PAF) [55,56]. *G. glabra* polysaccharide fractions exhibited immune-stimulating activity by stimulating macrophages and thereby raising the immune response [57]. N-acetyl muramoyl peptide is a glycyrrhizin isotope that shows in vitro activity toward the influenza virus, which is mediated by ceasing the virus’s reproduction [58]. Additionally, glycyrrhizic acid has been found to possess potential immunomodulatory activity by preventing virus multiplication and disrupting virus particles [59]. Several reports documented the anticancer efficacy of aqueous *G. glabra* extract and its related components in vitro [60]. For instance, glycyrrhetic acid was shown to promote the proapoptotic pathway by enhancing mitochondrial permeability transition, which, in particular, stimulates tumor cells apoptosis [61,62,63]. Methanolic licorice extract and its isolated compound, licocoumarone, were documented to stimulate the phosphorylation of BCl_2_ and halt the G2/M cycle in cancer cell lines and to induce human monoblastic leukemia U937 cells apoptosis. Furthermore, hydromethanolic root extract demonstrated antimutagenic activity by suppressing the formation of micronucleus and chromosomal abnormalities in the bone marrow cells of albino mice [64,65]. Recently, Yoon et al. [66] revealed that the novel retrochalcone component, licochalcone E that was isolated from *G. inflate* root extract, showed potent cytotoxic activity in comparison with the famous antineoplastic drugs (isoliquiritigenin and licochalcone A).

Hydro-alcoholic licorice rhizome extract was examined for its efficacy on the involuntary efficacy of the colon isolated from rats and these results revealed that the hydro-alcoholic extract altered the efficacy of colon motility through its synergism with β-adrenergic receptors only without affecting the α-adrenergic receptors [67,68]. The isoliquiritigenin compound isolated from licorice aqueous extract showed an effective relaxant effect by suppressing the contraction caused by different kinds of stimulants, such as BaCl_2_, carbamylcholine (CCh) and KCl [69,70]. Khoshnazar et al. [71] examined the mechanical activity of licorice rhizome extract on duodenal motility in the presence of β-adrenoceptor agonists, such as epinephrine; β-receptor antagonists, such as propranolol; muscarinic receptor agonists, such as acetylcholine; muscarinic receptor antagonists, such as atropine; or nitric oxide synthase (NOS) inhibitor such as (N-w-nitro-L-arginine methyl ester (L-NAME)). They revealed that licorice rhizome extract significantly reduced the duodenum contraction force induced by acetylcholine without affecting the β-adrenergic, cholinergic, and nitrergic pathways. Moreover, the mineralocorticoid activities of licorice have been found because of the cortisol metabolism inhibitors: 18 β-glycyrrhetinic acid and glycyrrhizin. Moreover, Hajirahimkhan et al. [72] reported the high estrogenic activity of *G. glabra* is attributed to the non-enzymatic conversion of isoliquiritigenin to liquiritigenin as well as partial estrogen agonist activity. 

**Figure 1:**
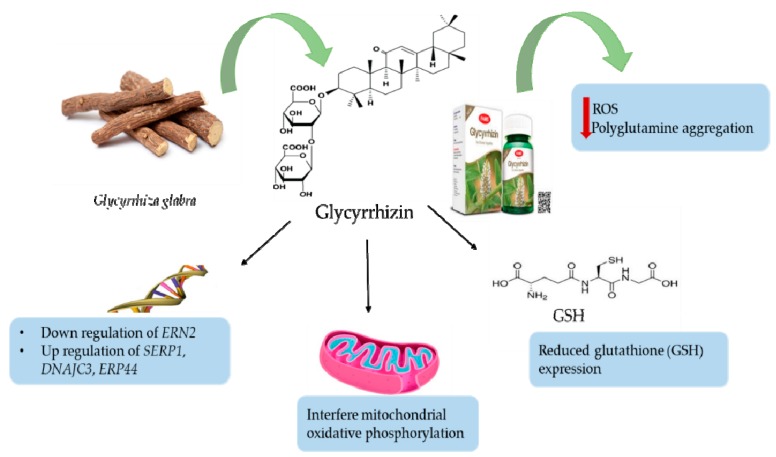
The effectiveness of licorice root extract and its related compound, glycyrrhizin in treating dementia and dementia associated with Alzheimer’s disease (AD). Photo source: https://famepharma.com/glycyrrhizin/.

Alzheimer’s disease (AD) is a genetically neurodegenerative disease, characterized by amnesia and cognitive disorders, such as depression, apathy, and psychosis that harm daily life [73,74]. Different *Glycyrrhiza* species were examined for their therapeutic efficacy as neurological protectors toward neurodegenerative disorders, such as dementia and AD, and this was attributed to their antioxidative activities, indicating that licorice extracts exhibited effectiveness toward different neurodegenerative diseases, such as taupathies and AD [75]. For instance, *G. inflata* extract has been documented to reduce spinocerebellar ataxia type 3 (SCA3) by increasing the nuclear factor erythroid 2-related factor 2-antioxidant-responsive elements (NFE2L2-ARE), coactivator 1α (PPARGC1A), and the peroxisome proliferator-activated receptor γ activities [76]. Glycyrrhizin as well as *G. inflata* extract inhibit ROS generation, cytotoxicity, and glutathione downregulation (GSH), the critical component of the brain’s antioxidative system, that are caused by 1-methyl-4-phenylpyridinium, a neurotoxic substance that intervenes with the mitochondrial oxidative phosphorylation [75,77]. The decreased GSH levels are the main cause of increased oxidative stress in dementia [78,79]. *Glycyrrhiza* extract activity on oxidative stress may be associated with the isoliquiritigenin effect on the function of mitochondria [80]. *Glycyrrhiza* acts by decreasing oxidative stress related to different types of dementia types by reducing brain cell damage, enhancing nerve cell function, and inhibiting memory weakness [81]. The licorice root extract and glycyrrhizin activities in the treatment of dementia and/or AD-related dementia are shown in Figure 1. 

Moreover, several reports documented that the memory-enhancing activity of licorice might be attributed to its anti-inflammatory effects and this discovery is consistent with findings that revealed the correlation between oxidative stress and inflammation [81,82]. It is worth noting that *Glycyrrhiza* has been used traditionally in many polyherbal formulations. For example, in Japan, a formulation, named yokukansan, which is traditional Japanese Kampo medicine, consisted of seven various plant species including *G. uralensis* [83]. Numerous plant components that show neuroprotective effects have been identified from *Glycyrrhiza* species listed in the yokukansan formulation, such as glycycoumarin, glycyrrhizin, isoliquiritigenin, and liquiritin [83]. Interestingly, the effectiveness of isoliquiritigenin in inhibiting N-methyl-D-aspartate (NMDA) receptors was similar to that demonstrated by memantine, an important synthetic drug against dementia [83,84], whereas the glycycoumarin neuroprotective effect can be due to its capacity to suppress the caspase-3 proapoptotic activity [83,85]. 

### 3.3. In Vivo Pharmacological/Biological Properties of G. glabra Extract and Its Metabolites 

Mi-Ichi et al. [47] revealed the antimalarial efficacy of chalcones as they found that chalcones completely eradicated *P. yoelii* parasite in mice without any toxic side effects, whereas Batiha et al. [49] exhibited the in vivo antipiroplasmic effect of chalcones against *Babesia* and *Theileria* parasites. Blatina [59] reported the antiviral efficacy of N-acetyl muramoyl peptide, the glycyrrhizin isotope that shows in vivo activities toward the influenza virus, which is mediated by ceasing the virus’s reproduction. Moreover, Nirmala and Selvaraj [39] reported the anti-inflammatory effect of hydro alcoholic *G. glabra* root extract against carrageenan-induced rat paw and they revealed that hydro alcoholic *G. glabra* root extract prevented leukocyte migration in a dose-dependent manner. The anti-inflammatory effect of *G. glabra* extract was attributed to the antioxidant potential of the glycyrrhizin, as inflammation includes oxidative injury these results were consistent with that shown by the standard indomethacin, the non-steroidal anti-inflammatory drug. Moreover, Adel et al. [53] documented that licorice can increase prostaglandin concentration in the digestive tract, thereby promoting the secretion of mucus from the stomach of male albino rats. In addition to that, licorice has shown anti-pepsin activity and has prolonged surface cell lifespan in the stomach. 

Root extract of *G. glabra* exhibited antidiabetic and lipid-lowering activities when administered to albino mice at low doses [86,87]. Antidiabetic activity of long-term treatment with glycyrrhizin was examined on non-insulin-dependent diabetic mice. The high percentage of glycyrrhizin in the diet lowered the blood glucose level seven weeks after the beginning of test feeding, while a low percentage did not suppress high levels of blood glucose in tested mice. On the other hand, water intake increased gradually in the control and low glycyrrhizin diet groups [88]. In the in vivo experiment, glycyrrhizin decreased the lipid and blood glucose levels by different mechanism of actions, particularly by inhibiting 11β-HSD. Lim et al. [89] reported that the intraperitoneal administration of glycyrrhizin at a dose of 50 mg/kg remarkably decreased 11β- HSD1 properties in the subcutaneous adipose tissue, liver, quadriceps femoris, kidneys, and abdominal muscle, while the kidneys only exhibited a remarkable decrease in 11β-HSD2 activities. Another study showed that the oral administration of 50 mg/kg of glycyrrhizin for seven days significantly reduced 11β-HSD1 activities in the liver only, while significantly decreasing the activities of 11β-HSD2 in both the livers and kidneys [90]. Eu et al. [91] revealed that high-calorie diet-fed rats treated with glycyrrhizin resulted in a remarkable decrease in their hepatic 11β-HSD1 activities with associated enhancements in lipid metabolism and gluconeogenesis reduction. The 11β-HSD1 inhibitory effect of glycyrrhizin was found to enhance lipid profiles and inhibit ectopic lipid storage, especially in the liver and visceral adipose tissue. All of these factors revealed that glycyrrhizin could be a potential therapeutic compound for the treatment and improvement of metabolic syndromes [92]. Furthermore, glycyrrhizin efficacy was observed on oxidative stress and diabetic alterations caused by streptozotocin (STZ), including iron-mediating free oxidation reactions caused by hemoglobin. Sen et al. [86] revealed that glycyrrhizin effectiveness was comparable to the well-known antidiabetic drug glibenclamide and they observed that the STZ diabetic efficacy was significantly stimulated by glycyrrhizin as it regulated glucose-intolerant behavior and blood glucose levels, enhanced glycohaemoglobin, cholesterol, and triglyceride levels, and reduced the level of serum insulin, including the numbers of pancreatic islet cell as well as pancreas and kidney tissue abnormalities due to diabetes. Moreover, glycyrrhizin administration affected the antioxidant enzymes, including serum fructosamine, superoxide dismutase, catalase, and malondialdehyde, in diabetic rats and restored them to their relevant values. It has been documented to prolong the thrombin and fibrinogen coagulation time and increase the duration of plasma recalcification in vivo [55]. 

Licorice extract has been shown to possess hepatoprotective activity against diclofenac-induced hepatotoxicity in vivo [93]. 18 β-glycyrrhetinic acid (glycyrrhizic acid aglycone) has hepatoprotective activity by preventing the generation of free radical and lipid peroxidation [94]. Moreover, glycyrrhizin has been reported to be used in the treatment of acetaminophen-induced hepatotoxicity and it acts by inhibiting CCl_4_-induced membrane lipid peroxidation [95]. Several reports documented the in vivo anticancer efficacy of aqueous *G. glabra* extract and its related components [64]. Shi et al. [96] revealed the uterine relaxant and analgesic efficacies of isoliquiritigenin, which was inhibited by L-NAME and indomethacin (COX-1/COX-2 inhibitor). They documented that isoliquiritigenin use could lead to a significant decrease in the writhing response caused by acetic acid and hot-plate tests in vivo. These results suggest that the spasmolytic effect of isoliquiritigenin on uterine contractions was attributed to Ca^2+^ channels, NOS, and cyclooxygenase (COX) inhibition [96]. The aqueous *G. glabra* roots and rhizomes extract exhibited an aphrodisiac efficacy in vivo and this activity is attributed to the presence of glycyrrhizin as the active ingredient [97]. Glycyrrhizin, liquiritigenin, and 18 β-glycyrrhetinic acid are the main components responsible for the antiallergic effects of licorice and they act by inhibiting Immunoglobulin E (IgE) production in ovalbumin-induced asthmatic mice and effectively prevented the scratching behavior and passive cutaneous anaphylactic reaction in mice. Therefore, they can be used to treat allergic diseases caused by IgE, such as dermatitis and asthma [98]. 18 β-glycyrrhetinic acid is a potent 11 β-HSD competitive inhibitor that decreases the effectiveness of 11 β-HSD, which leads to increased concentrations of peripheral and intrarenal corticosterone in vivo [99]. Interestingly, in vivo studies have investigated the memory-improving activity of *G. glabra* by testing the learning and memory in mice administered at 150 mg/kg. They showed great learning and memory enhancement efficacy in mice; however, its mode of action is not clear yet [100]. The oral administration of another *Glycyrrhiza* species, *G. glabra* extract, was reported to enhance the learning ability of mice [101]. This indicates that *G. glabra* extract is useful in improving the capacity for learning; however, its dose should be closely determined to inhibit the depressant effectiveness. Moreover, diammonium-glycyrrhizinate inhibited the cognitive and mitochondrial malfunctions caused by Aβ42 in vivo [101]. In conclusion, *Glycyrrhiza* extracts exhibit antioxidative and anti-inflammatory potential, and they regulate glutamate signaling and apoptosis. 

### 3.4. Clinical Efficacy of G. glabra Extract and Its Metabolites

Glycyrrhizin, the main active constituent of *G. glabra*, has shown potential antiviral efficacy, as virus-cell binding was inhibited and previously used to treat HIV-1 and chronic hepatitis C virus patients. Recent studies have examined the antiviral effectiveness of 6-azauridine, glycyrrhizin, pyraziofurin, mycophenolic acid, and ribavirin towards the FFM-1 and FFM-2 isolates of coronavirus in SARS-infected patients. Glycyrrhizin has been observed to be a potent drug in restraining viral reproduction and it also has shown a prophylactic effect [44,46,102]. In two clinical trials, a glycyrrhizin preparation, namely, Stronger Neo-Minophagen C, caused a remarkable decrease in alanine transaminase (ALT), gamma-glutamyl transferase (GGT), and aspartate transaminase (AST) levels, with increasing histological evidence of necrosis and inflammatory lesions in the liver [103]. Moreover, Stronger Neo-Minophagen C exhibited potent effects on the inhibition of liver inflammation and was effective in enhancing chronic hepatitis and liver cirrhosis [33]. 

Armanini et al. [104] investigated the ability of glycyrrhetinic acid and glycyrrhizin to bind to mineralocorticoid and glucocorticoid receptors. They revealed that the affinity of glycyrrhizin for mineralocorticoid receptors is less than that of aldosterone and its affinity for glucocorticoid receptors is less than that of dexamethasone. Therefore, the overconsumption of licorice can produce mineralocorticoid-like symptoms. The mineralocorticoid properties of licorice, the mineralocorticoid receptor agonist, and mild androgen synthesis inhibitor were suggested to decrease the incidence of the side effects associated with spironolactone, a mineralocorticoid receptor blocker [105,106]. 

Glycyrrhizin inhibits band 3 Tyr-phosphorylation caused by diamide and n-ethylmaleimide without affecting glutathione (GSH) downregulation [107]. Another study documented that this efficacy of glycyrrhizin is opposite from those of aldosterone, which enhances the changes caused by diamide. The protective activity of glycyrrhizin could be associated with its direct interaction at the plasma membrane level, but not due to the mineralocorticoid receptor, which inhibits membrane protein oxidation, and its glucocorticoid activity. These findings indicate that the pseudohyperaldosteronism and inflammatory effects of licorice are associated with its binding to the mineralocorticoid receptor and β-11HSD2 inhibition as well as with its anti-inflammatory and antiartherosclerotic activities that alter cellular membrane fluidity and oxidative stress modifications, and its estrogen- and glucocorticoid-like effects [21]. 

Although licorice has shown several other clinical applications due to its antiandrogen and estrogen-like activities, the effect of its active constituent, glycyrrhetinic acid, at the level of mineralocorticoid receptors and to β-11HSD2 is the main limitation to the medicinal use of licorice. Therefore, its use in association with spironolactone is important to avoid the major side effects, particularly in the treatment of polycystic ovarian syndrome (PCOS) to enhance the antiandrogen activity of spironolactone and limit its hypotensive properties. In PCOS patients, the mineralocorticoid activities of licorice can decrease the incidence of spironolactone diuretic side effects. Moreover, the combined effect of licorice with spironolactone results in a significant decrease in renin–aldosterone system activation as well as metrorrhagia [108,109]. Glycyrrhizin is suggested to enhance the integrity of red blood cell membranes against proteolytic and oxidative injury by inhibiting any changes caused by diamide and n-ethylmaleimide treatment, thus, preventing proteolytic injury [105,107]. The pharmacological action of phytochemicals isolated from licorice extracts is shown in Table 3.

### 3.5. Pharmacokinetics of G. glabra Extract and Its Metabolites

#### 3.5.1. In Animals

Glycyrrhizin and glycyrrhetic acid are known as 11β-HSD1 and 11β-HSD2 inhibitors, however, glycyrrhetic acid has shown a higher inhibiting effect on 11β-HSD1, leading to the conversion of active glucocorticoids into inactive glucocorticoids. Glucocorticoids have an important role in glucose-6-phosphatase and phosphoenolpyruvate carboxykinase regulation. Glycyrrhizin prevents 11β-HSD1 activity that leads to a reduction in active peroxisome proliferator-activated receptor agonism and glucocorticoids properties, which may be the cause of the increased expression of lipoprotein lipase in all tissues after glycyrrhizin administration [110].

#### 3.5.2. In Humans

Glycyrrhizin (glycyrrhizic acid), the main constituent of licorice, showed impaired oral bioavailability in humans and it was found at very low concentrations after oral administration of 100–1600 mg/kg. After oral administration licorice, glycyrrhizic acid is hydrolyzed to 18β-glycyrrhetic acid by the action of intestinal bacteria, which influences a specialized β-D-glucuronidase [23]. Glycyrrhetic acid pharmacokinetics, after oral ingestion, are more relevant than glycyrrhizic acid as they show 200–1000-times stronger 11 β-HSDs inhibition. Afterward, glycyrrhetic acid is rapidly absorbed and transferred by carrier molecules to the liver where it is metabolized by lysosomal β-D-glucuronidase to 3-mono-glucuronide 18β-glycyrrhetinic acid and sulfate conjugates, which subsequently re-degrade to glycyrrhetic acid and are reabsorbed, leading to a significant delay in terminal plasma clearance [111]. Neither glycyrrhizin nor 18 β-glycyrrhetinic acid have been documented to cumulate in tissues. The plasma clearance of glycyrrhizin and 18 β-glycyrrhetinic acid is only dose-dependent at high doses that exceed the serum protein binding saturation, while it is not dose-dependent at low doses below 120 mg in healthy people [19]. 

Previous studies documented that glycyrrhizin and 18 β-glycyrrhetinic acid pharmacokinetics could be affected by other phytochemical compounds present in licorice extracts. For instance, Isbrucker and Burdock [19] reported that glycyrrhizin and 18 βglycyrrhetinic acid concentrations, after aqueous licorice root extract administration to rats and humans, were low when compared to pure glycyrrhizin single therapy and significant variations were observed in the Tmax, areas under the plasma-time curve (AUC), and Cmax parameters. Moreover, Ploeger et al. [111] revealed that the plasma clearance of 18β-glycyrrhetinic acid decreases significantly in chronic hepatitis C and liver cirrhosis patients, indicating that the liver capacity is limited in 18 β-glycyrrhetinic acid metabolism and/or excretion in the bile. Notably, 18 β-glycyrrhetinic acid has also been documented to penetrate the placental barrier and this could be observed in the rat fetuses [111].

### 3.6. Dose, Side Effects, and Contraindications

The documented daily doses of licorice root for the treatment of ulcer and gastritis range between 1 to 15 g. However, administration of higher doses for long periods may increase the risk of hyperkalemia and cause serious increases in blood pressure and apparent mineralocorticoid excess [112,113,114]. Moreover, based on the in vivo and clinical evidence, Isbrucker et al. [19] suggested that the acceptable daily intake of glycyrrhizin is 0.015–0.229 mg/kg body weight/day. Vispute and Khopade [115] documented the half-maximal lethal concentration (LD_50_) values of glycyrrhizin in rats and mice as follows: LD_50_ values for the subcutaneous route of administration were 4–4.4 g/kg, 1.42-1.70 g/kg for the intraperitoneal route of administration, and 14.2-18.0 g/kg for the oral route of administration. Moreover, Omar et al. [23] reported that people with kidney or heart troubles are more prone to licorice and glycyrrhizin intoxication. Administration of high doses of glycyrrhizin causes pseudohyperaldosteronism, which makes a person hypersensitive to adrenal cortex hormones and this causes several adverse effects, such as heart attack, headaches, high blood pressure, fatigue, and water retention, which leads to leg swelling and other problems and it is contraindicated in pregnancy. In addition, licorice showed an estrogenic effect with abortifacient activity [114]. Glycyrrhizin is contraindicated for administration with oral contraceptives, hydrocortisone, and prednisolone [115]. Therefore, research towards the finding the optimum dose to prevent the adverse effects of plants and discovering new molecules with potent pharmacological effects are necessary in future. [116,117].

## 4. Conclusions

This review examined the medicinal properties and all the phytochemical molecules isolated from *Glycyrrhiza glabra*. Glycyrrhizic acid, 18-β-glycyrrhetinic acid, glycyrrhizin and licochalcones are the main constituents that have been isolated from *G. glabra* extracts. Pharmacologically, *G. glabra* and its main constituents possess antimicrobial, antiparasitic, antiviral, antitussive, immuno-enhancing, antioxidant, anti-inflammatory, and anticancer effects. Moreover, they show hepatoprotective, anticoagulant, antidiabetic, and spasmolytic activities. Glycyrrhizin, the main active constituent of *G. glabra*, is contraindicated for administration with oral contraceptives, hydrocortisone, and prednisolone. Administration of high doses of glycyrrhizin causes pseudoaldosteronism that may leads to several adverse effects. More detailed studies regarding the mechanism of action of extracts and compounds, and the determination of effective dose, interaction and side effects are necessary.

## Figures and Tables

**Table 1:** Scientific classification of *G. glabra*.

Taxonomy	
**Realm**	Plantae
**Division**	Magnoliophyta
**Class**	Magnoliopsida
**Order**	Fabales
**Family**	Fabaceae
**Genus**	*Glycyrrhiza*
**Species**	*Glycyrrhiza glabra* L.

**Table 2:** International Union of Pure and Applied Chemistry (IUPAC) name and chemical formula of bioactive molecules isolated from *G. glabra.*

Compound	IUPAC Name	Chemical Formula	Compound	IUPAC Name	Chemical Formula
**Glycyrrhizin**	(3β,20β)-20-carboxy-11-oxo-30-norolean-12-en-3-yl 2-O-β-D-glucopyranuronosyl-α-D-glucopyranosiduronic acid	C_42_H_62_O_16_	**Glabridin**	4-[(3*R*)-8,8-Dimethyl-3,4-dihydro-2*H*,8*H*-pyrano [2,3-*f*]chromen-3-yl]-1,3-benzenediol	C_20_H_20_O_4_
**Glycyrrhizic acid**	(2*S*,3*S*,4*S*,5*R*,6*R*)-6-[(2*S*,3*R*,4*S*,5*S*,6*S*)-2-[[(3*S*,4*aR*,6*aR*,6*bS*,8*aS*,11*S*,12*aR*,14*aR*,14*bS*)-11-carboxy-4,4,6*a*,6*b*,8*a*,11,14*b*-heptamethyl-14-oxo-2,3,4*a*,5,6,7,8,9,10,12,12*a*,14*a*-dodecahydro-1*H*-picen-3-yl]oxy]-6-carboxy-4,5-dihydroxyoxan-3-yl]oxy-3,4,5-trihydroxyoxane-2-carboxylic acid	C_42_H_62_O_16_	**Glabrene**	8-(7-hydroxy-2H-chromen-3-yl)-2,2-dimethylchromen-5-ol	C_20_H_18_O_4_
**Isoliquiritigenin**	(*E*)-1-(2,4-Dihydroxyphenyl)-3-(4-hydroxyphenyl)prop-2-en-1-one	C_15_H_12_O_4_	**Licocoumarin A**	3-[2,4-dihydroxy-3-(3-methylbut-2-enyl)phenyl]-7-hydroxy-8-(3-methylbut-2-enyl)chromen-2-one	C_25_H_26_O_5_
**Licochalcone A**	(*E*)-3-[4-Hydroxy-2-methoxy-5-(2-methylbut-3-en-2-yl)phenyl]-1-(4-hydroxyphenyl)prop-2-en-1-one	C_21_H_22_O_4_	**18-β-Glycyrrhetinic acid**	(2*R*,4*aS*,6*aS*,6*bR*,8*aR*,10*S*,12*aS*,14*bR*)-10-hydroxy-2,4*a*,6*a*,6*b*,9,9,12*a*-heptamethyl-13-oxo-3,4,5,6,6*a*,7,8,8*a*,10,11,12,14*b*-dodecahydro-1*H*-picene-2-carboxylic acid	C_30_H_46_O_4_
**Liquiritigenin**	(2*S*)-7-Hydroxy-2-(4-hydroxyphenyl)-2,3-dihydro-4*H*-chromen-4-one	C_15_H_12_O_4_	**Liquiritin**	(2*S*)-7-hydroxy-2-[4-[(2*S*,3*R*,4*S*,5*S*,6*R*)-3,4,5-trihydroxy-6-(hydroxymethyl)oxan-2-yl]oxyphenyl]-2,3-dihydrochromen-4-one	C_21_H_22_O_9_
**Prenyllicoflavone A**	7-Hydroxy-2-[4-hydroxy-3-(3-methyl-2-buten-1-yl)phenyl]-6-(3-methyl-2-buten-1-yl)-4H-1-benzopyran-4-one	C_25_H_26_O_4_	**Kanzonol R**	3-[2-hydroxy-4-methoxy-3-(3-methylbut-2-enyl)phenyl]-5-methoxy-3,4-dihydro-2*H*-chromen-7-ol	C_22_H_26_O_5_
**α-Terpineol**	2-(4-Methylcyclohex-3-en-1-yl)propan-2-ol	C_10_H_18_O	**Glisoflavone**	3-[3,4-dihydroxy-5-(3-methylbut-2-enyl)phenyl]-7-hydroxy-5-methoxychromen-4-one	C_21_H_20_O_6_
**Shinpterocarpin**	(2*R*,10*R*)-17,17-dimethyl-3,12,18-trioxapentacyclo[11.8.0.0^2,10^.0^4,9^.0^14,19^]henicosa-1(13),4(9),5,7,14(19),15,20-heptaen-6-ol	C_20_H_18_O_4_	**Isoangustone A**	3-[3,4-dihydroxy-5-(3-methylbut-2-enyl)phenyl]-5,7-dihydroxy-6-(3-methylbut-2-enyl)chromen-4-one	C_25_H_26_O_6_
**1-Methoxyficifolinol**	(6*aR*,11*aR*)-1-methoxy-2,8-bis(3-methylbut-2-enyl)-6*a*,11*a*-dihydro-6*H*-[1]benzofuro[3,2-c]chromene-3,9-diol	C_26_H_30_O_5_	**2,3-Butanediol**	Butane-2,3-diol	C_4_H_10_O_2_
**Licoriphenone**	1-(2,4-dihydroxyphenyl)-2-[6-hydroxy-2,4-dimethoxy-3-(3-methylbut-2-enyl)phenyl]ethanone	C_21_H_24_O_6_	**Semilicoisoflavone B**	5,7-dihydroxy-3-(8-hydroxy-2,2-dimethylchromen-6-yl)chromen-4-one	C_20_H_16_O_6_
**Licoarylcoumarin**	3-(2,4-dihydroxyphenyl)-7-hydroxy-5-methoxy-8-(2-methylbut-3-en-2-yl)chromen-2-one	C_21_H_20_O_6_	**Licopyranocoumarin**	7-(2,4-dihydroxyphenyl)-2-(hydroxymethyl)-5-methoxy-2-methyl-3,4-dihydropyrano[3,2-g]chromen-8-one	C_21_H_20_O_7_
**Furfuraldehyde**	Furan-2-carbaldehyde	C_5_H_4_O_2_	**Tetramethyl pyrazine**	tetramethyl pyrazine-2,3,5,6-tetracarboxylate	C_12_H_12_N_2_O_8_

**Table 3:** Chemical components responsible for licorice efficacy.

Activities	Chemical Component	Category	References
Antiulcer	Glycyrrhizic acid and glabridin, glabrene	Triterpenoid saponin and flavonoid	[50]
Antimycobacterial	Isoliquiritigenin	Flavonoid	[42]
Uterine relaxant and analgesic	Licocoumarin, licochalcone, isoliquiritigenin, and glabridin	Coumarin and flavonoids	[97]
Antioxidant	Glabridin	Flavonoid	[51,52]
Memory-enhancing activity	18-β-glycyrrhetinic acid	Triterpenoid	[82]
Corticosteroidal activity	Liquiritigenin, glycyrrhizin, and 18-β-glycyrrhetinic acid	Flavonoid and triterpenoid saponin	[54]
Antiallergic	Glycyrrhizin	Triterpenoid saponin	[98]
Hepatoprotective	Liquiritoside and glycyrrhetic A	Flavonoid and triterpenoid saponin	[95]
Anti-inflammatory	Glycyrrhizin and glycyrrhetic A	Flavonoid	[26,60]
Anticancer	Licochalcone A	Flavonoid	[66]
Antimalarial	Glycyrrhizin, licochalcone, glycyrrhetinic acid	Flavonoid and triterpenoid	[47]
Antiviral activity	Glycyrrhizin and 18-β-glycyrrhetinic acid	Triterpenoid saponin	[45,46]
Antihyperglycemic	Glycyrrhizin	Triterpenoid saponin	[87]
Antitussive activity	Isoliquiritigenin and glycyrrhizin	Flavonoid and triterpenoid saponin	[35]
Immunostimulating activity	Glycyrrhizin	Triterpenoid saponin	[82]
Anti-HIV	Glycyrrhizin	Triterpenoid saponin	[45]
Muscle relaxant	Glabridin	Flavonoid	[71]
Antimicrobial	Liquiritigenin and glabrene	Flavonoid	[25,41]

## Abbreviations

HPLC	high-performance liquid chromatography
*G. glabra*	*Glycyrrhiza glabra*
IUPAC	International Union of Pure and Applied Chemistry
11 beta-HSD2	11 beta-hydroxysteroid dehydrogenase
HIV	human immunodeficiency virus
ROS	reactive oxygen species
MAO	monoamine oxidase
GAMG	glycyrrhetinic acid monoglucuronide acetylated
SARS	severe acute respiratory syndrome
DGL	deglycyrrhizinated licorice
STZ	streptozotocin
PAF	platelet aggregating factor
CCh	carbamylcholine
L-NAME	N-w-nitro-L-arginine methyl ester
AD	Alzheimer’s Disease
SCA3	spinocerebellar ataxia type 3
NFE2L2-ARE	nuclear factor erythroid 2-related factor 2-antioxidant-responsive elements
PPARGC1A	coactivator 1α
AUC	areas under the plasma-time curve
LD_50_	half-maximal lethal concentrations.
